# Comparison of Microfluidic Synthesis of Silver Nanoparticles in Flow and Drop Reactors at Low Dean Numbers

**DOI:** 10.3390/mi16010075

**Published:** 2025-01-10

**Authors:** Konstantia Nathanael, Nina M. Kovalchuk, Mark J. H. Simmons

**Affiliations:** 1 School of Chemical Engineering, University of Birmingham, Birmingham B15 2TT, UK; cxn782@student.bham.ac.uk (K.N.); m.j.simmons@bham.ac.uk (M.J.H.S.); 2 Department of Mechanical Engineering and Materials Science and Engineering, Cyprus University of Technology, Limassol 3036, Cyprus

**Keywords:** microfluidic synthesis, silver nanoparticles, continuous flow reactors, drop reactors

## Abstract

This study evaluates the performance of continuous flow and drop-based microfluidic devices for the synthesis of silver nanoparticles (AgNPs) under identical hydrodynamic and chemical conditions. Flows at low values of Dean number (De < 1) were investigated, where the contribution of the vortices forming inside the drop to the additional mixing inside the reactor should be most noticeable. In the drop-based microfluidic device, discrete aqueous drops serving as reactors were generated by flow focusing using silicone oil as the continuous phase. Aqueous solutions of reagents were supplied through two different channels merging just before the drops were formed. In the continuous flow device, the reagents merged at a Tee junction, and the reaction was carried out in the outlet tube. Although continuous flow systems may face challenges such as particle concentration reduction due to deposition on the channel wall or fouling, they are often more practical for research due to their operational simplicity, primarily through the elimination of the need to separate the aqueous nanoparticle dispersion from the oil phase. The results demonstrate that both microfluidic approaches produced AgNPs of similar sizes when the hydrodynamic conditions defined by the values of De and the residence time within the reactor were similar.

## 1. Introduction

According to a 2022 report released by Allied Market Research [1], the global market for microfluidic technologies is estimated to reach USD 158.1 billion by 2031. Their rapid growth is driven by numerous advantages over larger-scale systems, such as precise control of hydrodynamic and thermal conditions, short diffusion distances, and the possibility of optimisation through high-throughput testing using minimal amounts of reagents and energy. Microfluidic technologies have been successfully used in a range of applications, including enhanced oil recovery, water treatment, catalysis, chemical synthesis, diagnostics, drug discovery, and DNA analysis [2,3,4,5].

Continuous [6] and segmented [7] flows are two primary operational approaches used in microfluidic synthesis. Continuous flow systems can be either single-phase or multiphase. In a single-phase process [8], reagents are dissolved in the same solvent (usually water) and supplied by separate inlet channels which meet at a junction where the reaction initiates. The reaction then progresses further in the outlet channel. For the microfluidic synthesis of nanoparticles (NPs), the reagents include precursors, reducing agents, and stabilisers. Their mixing triggers a reaction between the precursor and the reducing agent with the consequent nucleation and growth of nanoparticles. The multiphase continuous flow process, usually including two phases [9], is often used for the fabrication of polymeric NPs. The polymer is initially dissolved in a good solvent, which meets at a junction with a miscible anti-solvent, and the two streams flow together. The mass transfer of anti-solvent results in the nanoprecipitation of polymer forming nanoparticles [10].

Drop-based microfluidics (segmented flow) employ immiscible liquid phases, one of which is dispersed in the other as spherical or pancake-shaped droplets or elongated plugs. Reagents can be present either in both phases or only in one, usually the dispersed phase, while the continuous phase is used to separate each drop reactor. Examples of the former approach are the synthesis of hydrogel microparticles by external gelation [11] and biocatalysis [12]. In the synthesis of metallic NPs, including AgNPs, the dispersed phase is usually a solution of reagents in water [13] or in an ionic liquid [14], whereas the continuous phase is an inert oil which transports the droplets through the channels. For reactions carried out within the droplets of the dispersed phase, there are two main operational approaches. In the first approach, the reagents are combined together during the droplet formation phase [15], while, in the second approach, the reagents are initially contained in separate droplets, which then collide and merge within the channel to initiate mixing and trigger chemical reactions [16]. Various methods including cross-flow [17], flow focusing [18], and co-flow [19] are used for drop formation in microfluidic systems, primarily within the squeezing/dripping and jetting regimes [20]. Monodisperse drops are most commonly formed in the dripping regime [21], demonstrating its suitability for drop generation in microfluidics. The drop size in microfluidic systems is influenced by the interfacial tension, the viscosity ratio between the continuous and dispersed phases, and the flow rates of these phases. These parameters can be experimentally adjusted to achieve the desired drop size [22].

The rapid growth in nanoparticle usage is driven by their unique size-dependent properties when compared with bulk materials [23]. Applications include optical sensors [24,25,26], substrates for surface-enhanced Raman scattering (SERS) [27], photocatalysis [28,29,30], electronics [31,32,33], and energy storage [34]. All these applications rely on the precisely tuned properties of the NPs used. Industry-relevant bottom–up methods for NP production include aerosol-based processes, gas condensation, arc discharge, laser ablation, plasma processes, chemical vapour deposition, sol–gel, and solvothermal methods [35]. However, for the metallic NPs considered in this study, the main method of production is batch liquid-phase synthesis based on the reduction of metal precursors in dilute solutions [35]. As a consequence, many previous studies focused on the comparison of batch reactors with microfluidic devices [36,37,38]. They were motivated by the fact that batch reactors typically process larger volumes of reactants and consume more energy per experiment when compared to microfluidic systems. Thus, using microfluidics can minimise resource consumption and significantly reduce the amount of undesired by-products and waste, thereby reducing the environmental impact of process optimisation for nanoparticle synthesis [39] if the process condition can be upscaled to batch reactors or larger continuous flow reactors. There has been limited exploration of continuous- flow versus segmented flow reactors for the synthesis of polymeric NPs [40], with no studies related to the synthesis of metallic NPs. To fill this gap, this study compares the synthesis of AgNPs in continuous flow and drop microfluidic devices.

A summary of relevant experimental studies on the synthesis of AgNPs using microfluidic technology can be found in a recent review by Nathanael et al. [41]. The formation of silver nanoparticles (AgNPs) involves three main stages: (i) silver ions are reduced to silver atoms; (ii) nucleation occurs, where the smallest stable clusters are formed; and (iii), these clusters grow into nanoparticles [42]. During AgNP synthesis, the reduction stage occurs rapidly, in less than 200 milliseconds, as noted by Polte et al. [43]. Ensuring the uniform mixing of the silver precursor with other reactants is thus critical for achieving consistent reaction conditions during this short, initial phase.

Mixing in batch reactors relies on mechanical agitation or stirring, whereas microfluidic systems exploit laminar flow, enabling mixing through diffusion, passive convective mixing, or alternatives such as acoustic or electric methods [44]. Passive convective mixing in microfluidics relies on the channel design and geometric structure of the microfluidic channels to enhance fluid mixing without requiring external energy sources. Different studies have placed obstacles within the channel to disrupt the laminar flow and induce vortices that enhance mixing [45,46]. Utilizing spiralling channels induces chaotic advection and the continuous formation of Dean vortices, facilitating efficient mixing through the repeated folding of fluid streams [47,48,49,50].

Continuous flow microfluidic reactors are easy to fabricate and operate, but they have some notable disadvantages [51]. The fluid flow creates a parabolic velocity profile; the speed of the fluid is higher in the centre and reaches zero at the wall (no-slip condition). This non-uniform velocity distribution leads to a consequent non-uniformity in fluid residence time. Additionally, the slow motion near the channel walls can cause particle deposition and fouling or blockage of the channel. The performance of continuous flow reactors can be improved by adopting strategies to reduce fouling [52] and incorporating spiralling and serpentine channels to promote radial mass transfer, which leads to better mixing and a narrower range of residence times [53,54]. Utilizing drop-based methods is an alternative approach to overcome the limitations of continuous flow reactors. There is, however, a considerable disadvantage to using drop reactors, since the process then requires an additional step of downstream separation of particle dispersion from the continuous oil phase.

The mixing conditions between the flow and drop reactor are very different. There is no recirculation in a straight-flow reactor, whereas there is always recirculation in a drop moving in a straight channel [55] which facilitates mixing and improves reaction conditions [56,57]. The vortices formed inside the drop moving in a rectangular channel are symmetrical, and, therefore, they cannot mix the drop content across the middle planes parallel to the channel walls. However, once the symmetry of reagent distribution is disturbed, for example, when a drop moves around a corner in a curved or serpentine channel, further mixing occurs rapidly.

To understand the influence of the internal circulation inside the drop reactor upon the NP size, a comparison between flow and drop reactors should be performed under conditions of a small Dean number, where the contribution of the vortices forming inside the drop compared to the additional mixing inside the reactor should be most noticeable. For continuous flow reactors with cylindrical channels between 5 and 15 mm in diameter, the best mixing is observed at De = 10 [58]. For a microfluidic device, a considerable effect on AgNP size and size distribution has been observed for De ≥ 2 [53], with a very broad size distribution at De = 2 and narrow distribution at De ≥ 7. The analysis of the data presented in [59,60] for the same set of reagents as this study shows that the change from the straight channel (De = 0) to the coiled channel with a small De ≤ 0.34 resulted in a statistically significant decrease in AgNP size in 69% of cases; in the remaining 31%, the difference in size was statistically insignificant. Therefore, in this study, values of De < 1 were used to better illustrate the difference between the drop and flow reactors.

A comparison between continuous flow and drop-based microfluidic devices under identical hydrodynamic and chemical conditions is important to provide a comprehensive understanding of the advantages and limitations of each approach, specifically to determine whether the performance differences are due to the design of the microfluidic systems rather than variations in the experimental operating conditions.

## 2. Materials and Methods

### 2.1. Chemical Reagents

The synthesis of AgNPs was conducted using silver nitrate ($Ag{NO}_{3}$ (SN)) as the precursor and tannic acid ($C_{76}H_{52}O_{46}$ (TA)) as a weak reducing agent in combination with trisodium citrate $({Na}_{3}C_{6}H_{5}O_{7}\cdot 2H_{2}O$ (TC)), which served in the reaction as both a reducing and stabilising agent.

In this chemical protocol, the concentrations of SN and TA were 0.92 mM and 0.123 mM, respectively, while the concentration of TC ranged from 1.91 to 3.82 mM. This experimental procedure was adopted from the work of Kašpar et al. [15], with adjustments made to the concentrations of TA and TC.

Although the concentration of TA was smaller than that of SN, TA was chosen to be in an excess due to its reducing ability. Each molecule of TA can effectively reduce up to 20 molecules of SN, whereas trisodium citrate can only reduce 4 silver ions [61,62]. For example, the molar ratio of TA to SN is 0.05 based on stoichiometry, but, in the investigated experimental case, it was 0.13, indicating that TA had been set in excess. The pH of the aqueous solution of tannic acid and trisodium citrate was set to either 7 or 12 using 0.1 M NaOH.

Silver nitrate (99+% (AgNO_3_)), tannic acid (C_76_H_52_O_46_), trisodium citrate dihydrate (Na_3_C_6_H_5_O_7_·2H_2_O), and sodium hydroxide (NaOH) were purchased from Alfa Aesar (Haverhill, MA, USA). Silicone oils with viscosities of 5 cSt (4.6 mPa s) and 20 cSt (19 mPa s) were purchased from Merck (Darmstadt, Germany). All aqueous solutions were prepared in double-distilled water produced by a water still Aquatron A 4000 D (Stuart).

### 2.2. Drop Microfluidic Device

The most common fabrication routes for microfluidic devices involve photolithography, soft lithography, micromachining and 3D printing, where microchannels and structures are created on substrates like glass, silicon, or polymers [63].

**Figure 1:**
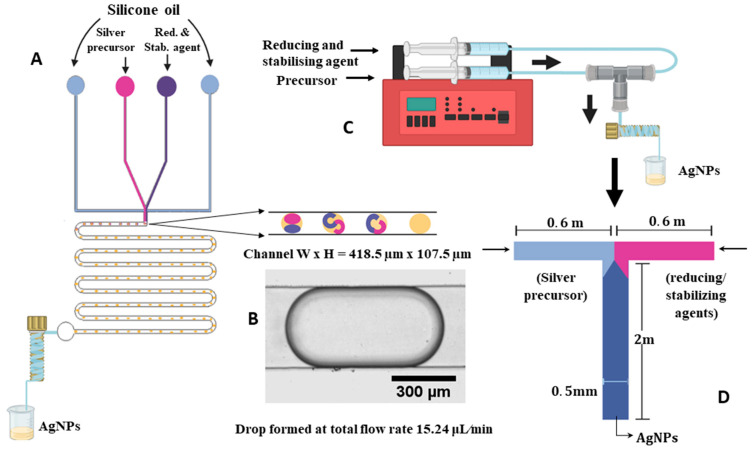
(**A**) Flow-focusing microfluidic device showing the formation of drops inside the microfluidic channels where the chemicals react to form AgNPs; (**B**) a typical drop in the channel; (**C**) experimental set up or AgNP synthesis in the flow device; and (**D**) schematic diagram of Tee-junction microfluidic device.

The drop microfluidic device used in this study (Figure 1A) consisted of two main components: a plain glass micro-slide measuring 75 mm ×50 mm from Corning^®^, coated with a layer of PDMS, and a tightly attached geometry made from PDMS by standard soft lithography [64].

The PDMS components, including the glass coating and the geometry structure, were prepared using a silicone elastomer base and a curing agent (Sylgard 184, Dow Corning (Midland, MI, USA)) in a ratio of 10:1. The mixture was blended in a plastic cup, and then it was placed in a vacuum chamber to remove air bubbles. The coated glass plate was prepared by adding a small quantity of PDMS onto the centre of the micro-slide, followed by even distribution using a POLOS^TM^ spin-coater (1000 rpm); the plate was then heated on a hot plate at 95 °C for 40 min and left to cool.

The device geometry was designed using AUTOCAD and then utilised to make a mould on a silicon wafer through photolithography [65] using SU-8 2075 photoresist. PDMS prepared as described above was then poured onto the wafers and put in a vacuum to remove bubbles. After this, the PDMS was cured in a furnace at 70 °C for 2 h. The resulting geometry was precisely cut out; holes were punched for the inlet and exit tubes and attached to the plate after the treatment of both surfaces by a corona discharge pen from OPlasma. Once bonded, the device was placed on a hot plate for 20 min at 95 °C. Novec 1720 (3M) was flushed through the device to hydrophobise the channels, followed by purging with air and oil to remove any residuals of Novec from the microchannels.

The PDMS channels were connected to 500μm inner diameter Tygon^®^ microbore tubing from Cole-Parmer and 5 mL syringes (Fisherbrand) were used to load the solutions. The flow rates were controlled with a World Precision Instrument syringe pump. The outlet tube had a length of 1.47 m and was coiled on a 3D-printed helical device with a 3 mm diameter to promote further mixing of the reagents. The details on the calculation of the outlet tube for the drop device are provided in Section 3.2.

Discrete drops were generated within a flow-focusing microfluidic device where the continuous phase consisted of silicone oil and the dispersed phase comprised the reagents. For the drop microfluidic device (see Figure 1A), two separate solutions were utilised as disperse phases (one containing SN and the other TA and TC), while the continuous phase was supplied through two side inlets to form drops by hydrodynamic flow focusing.

Drops with the reagents were formed within the oil phase and were carried along the microchannel. A snapshot of a typical drop used as a reactor is shown in Figure 1B. A drop size larger than the channel width was chosen to intensify convective mixing inside the drops. To further improve mixing, the flow direction was periodically reversed by using turnings with a radius of curvature of 688 µm. Mixing visualisation within the drop was carried out by adding Nigrosin dye (Alfa Aesar) in a concentration of 5 g/L to one of the aqueous phases. Drop formation and movement were observed using a Nikon Eclipse Ti2-U microscope set to 20× magnification with a resolution of 1 μm/pixel to ensure the lack of blockage of the microfluidic channels, backflow in the inlet channels, channelling, drops sticking to walls, or drop coalescence. Preliminary experiments were conducted to eliminate these issues and determine the optimal continuous phase for the investigated case. Vegetable and silicone oil were investigated for their ability to form stable drops. While vegetable oil had a good performance in producing stable drops, its surfactant content raised concerns regarding potential interaction with other chemicals and the risk of non-uniform particle distribution.

Initially, silicone oil with a kinematic viscosity of 20 cSt and a density of 0.95 g/mL was used as the continuous phase, which led to the formation of more stable drops of the dispersed phase due to its higher viscosity. However, in the end, to facilitate the separation of AgNPs from the oil phase before characterisation, silicone oil with a kinematic viscosity of 5 cSt and a density of 0.91 g/mL was used as the continuous phase due to its larger density difference from water (0.999 g/mL).

### 2.3. Tee-Junction Continuous Flow Device

The Tee-junction continuous flow device (Figure 1C,D) was composed of two inlets (length of 0.6 m each) of 500 μm inner diameter (ID) polytetrafluoroethylene (PTFE) cylindrical tubing from Cole-Palmer. The aqueous solutions of the reagents were supplied by two separate inlet channels meeting at a Tee tubing junction (0.020″ Thru-Hole) (Upchurch Scientific), whereas the reaction was carried out in the outlet channel of 2 m length (ID = 500 μm).

The inlet flow rates were controlled using a syringe pump from the World Precision Instrument-AL-4000 equipped with two 5 mL plastic syringes (Fisher). Using a two-syringe pump ensured identical flow rates for both inlets. The flow rate referred to in the below discussion is defined as the flow rate for one inlet. Hence, Q denotes the flow rate of each individual inlet and the total ($Q_{total}=2\times Q$). The outlet tubing was coiled using a 3D-printed helix device with a diameter of 3 mm to improve the mixing of the reagents.

### 2.4. Characterisation Method

Particle size distribution (PSD) was measured for both the continuous and drop-based microfluidic devices in triplicate, across three independent experiments, using the Zetasizer Nano series (Malvern Panalytical, Malvern, UK) with the refractive index of the AgNPs set to 0.135 [66]. A previous study [59] showed that the AgNP sizes obtained from the Zetasizer using this refractive index were in good agreement with the results from transmission electron microscopy (TEM).

## 3. Results

### 3.1. Drop Reactor

**Figure 2:**
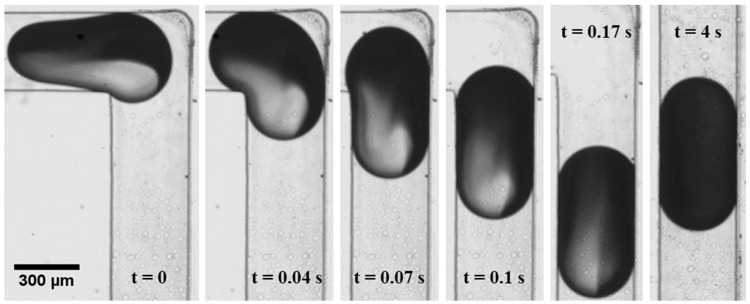
Mixing within a drop in the microfluidic device.

The drop reactor used in this study operates through the generation of discrete drops containing reactants within a continuous silicone oil phase using flow focusing. As these drops flow through the reactor, chemical reactions occur within them (see Figure 1A). When a drop is moving in a straight channel, mixing occurs between the front and rear of the drop, but there is no mixing across the channel width. In the dye experiments, the difference in colour between the two parts of the drop is visible immediately after drop formation (Figure 2, t = 0), although some degree of mixing has already occurred at this stage due to device non-ideality. After turning the corner, the dye distribution in the drop changes, so that more dye is present at the rear of the drop than at the front, facilitating mixing in the next straight part of the channel (Figure 2, t ≤ 0.17 s). After the drop has turned around the second corner, the colour distribution in the drop becomes visually uniform, confirming good mixing (Figure 2, t = 4 s).

The choice of flow rates for the drop reactor used in this study is discussed in Section 3.2. Two total flow rates, 15.24 µL/min and 30.48 µL/min, were used with a flow rate ratio between the continuous and dispersed phases set to 2:1. The drops formed at both flow rates were larger than the channel width, with a length of 720 ± 48 µm at 15.24 µL/min and 635 ± 18 µm at 30.48 µL/min.

As shown in Table 1, the size of the AgNPs synthesised in the drop-based reactor was mainly influenced by the pH of the solution and the concentration of TC. A change in the total flow rate from 15 to 30 µL/min did not result in a statistically significant change in the particle size, although a considerably narrower PSD was observed at 30 µL/min for a TC concentration of 1.91 mM and pH 12. The size of the AgNPs was seen decreasing as the pH of the solution and concentration of TC increased. These results agree with previous studies, which examined the effect of the pH [67], stabilizing agent [68], and residence time [69]. For example, for lower concentrations of the stabilising agent, the surface coverage was probably incomplete, enabling further particle growth. Also, further growth of particles can happen at lower flow rates because of a higher residence time in the reaction channel. Under alkaline conditions, the reducing ability of citrate is improved, leading to a faster reduction and smaller particles [70,71].

To compare the outcomes of the synthesis between the drop reactor and the Tee-junction continuous flow microfluidic device, the same residence time and hydrodynamic and chemical conditions needed to be achieved, using the same concentrations of reagents and pH of the solutions. The continuous flow device employed (see Figure 1C,D) operated with homogeneous streams, where enhancing the mixing efficiency relied primarily on coiling the outlet tube around a helical device, as detailed in Section 2.3.

### 3.2. Comparison of Continuous Flow and Drop Microreactors

Matching hydrodynamic conditions was achieved by matching the value of the Dean number (De) (Equation (1)). The Dean number was chosen because it provides the best representation of mixing conditions.

(1) De=ρudμd2Rc

where d is the inner diameter of the microchannel, $R_{c}$ is the radius of the helix curvature, *ρ* is the fluid density, u is the average velocity of the fluid, and μ is the viscosity of the fluid.

For the continuous flow device using a 3 mm helix and a 2 m outlet tube, operating at a total flow rate of 20 μL/min, the De is 0.34. The cross-section of the drop microfluidic device was rectangular; therefore, the hydraulic diameter was used as the inner diameter of the microchannel in Equation (1) to find the average velocity in the drop device, corresponding to De = 0.34. The hydraulic diameter (Equation (2)) [72] was calculated as follows:

(2) $$D_{h}=4\frac{A}{P}=2\frac{WH}{W+H}$$

where A represents the area of the channel cross-section, P denotes the perimeter of the channel, and W and H are the width and height of the channel, respectively, measured as 418.5 μm and 107.5 μm for the investigated channels. Subsequently, the total flow rate for the drop device was determined to be 15.24 μL/min.

**Figure 3:**
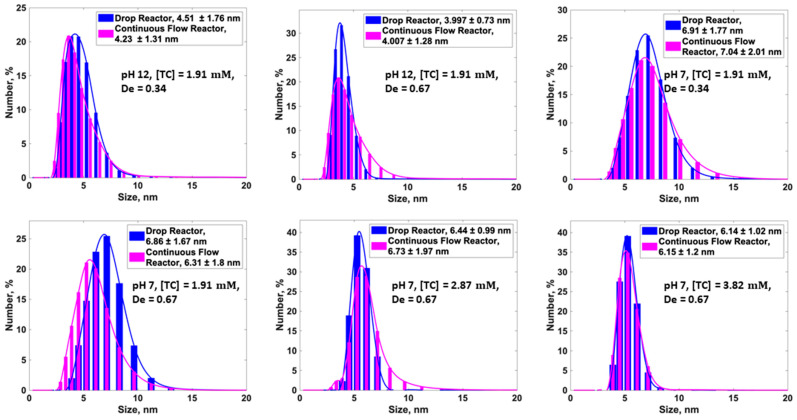
Comparison of the PSD obtained from drop- and continuous flow reactors under the same hy-drodynamic and chemical conditions.

Preliminary experiments indicated that a 2:1 ratio of the continuous and dispersed phases was effective for controlling the drop size and the distance between drops. Thus, the flow rates for the continuous and dispersed phases were set to 10.16 μL/min and 5.08 μL/min, respectively. The residence time in the continuous flow reactor at a total flow rate of 20 μL/min was thus estimated as 19.63 min. To match this residence time, an exit tube of 1.47 m length was attached to the exit of the drop device and coiled on the same helix as the continuous flow device. The cross-sectional area of the output tube was larger than that for the channel in the drop device; thus, the Dean number in the output tube was also smaller. To understand the impact of the variation in the Dean number on the NP size, a total flow rate of 30 μL/min was also examined for the drop reactor, and the effect of this change in flow rate was found to be negligible (Table 1). Upon analysing the data, it was found that using the same De (0.34 and 0.67) for both the continuous flow device and the drop reactor revealed no significant difference in the size of the synthesised AgNPs, as shown in Figure 3. The data show that, under the same chemical conditions, residence time, and Dean number, the size of the AgNPs and PSD was the same. The number distribution is used in Figure 3 instead of the intensity distribution, as it is in better agreement with the particle size distribution measured independently from the TEM images in our previous study using a flow reactor [59]. The intensity distribution revealed another peak at a particle size around 10 times larger, caused by aggregates which were rare and not observable in the TEM images. It should be stressed that the intensity distribution also showed very similar patterns for the AgNPs synthesised in the drop and flow reactors.

The synthesis of AgNPs and many other metallic NPs is best described by the Finke–Watzky kinetic model, which suggests that the best conditions for particle synthesis are achieved when mass transfer does not affect synthesis kinetics [73]. It was also shown in [60] that the improved mixing results in a smaller particle size for AgNP synthesis, using the continuous flow reactor employed in this study. Therefore, it can be suggested that, to keep the particle size distribution similar for different chemical protocols, it is important to change the relative rate of mixing in line with changes in reaction kinetics, i.e., using a stronger reducing agent will require faster mixing (a larger Dean number).

The continuous flow microfluidic concept used in this study can be extended to microwave (MW)-assisted reaction chambers. The synthesis of metal NPs like Ag, whose growth is highly sensitive to the reaction conditions, can greatly benefit from the rapid and precise heating provided by MW irradiation [74]. Integrating microwave energy into a continuous flow device can significantly increase the reaction kinetics and enhance the reaction rates and efficiency of AgNP synthesis in continuous flow systems, especially under conditions of fast mixing. This approach can be used to scale-up production but also ensure better uniformity of the AgNPs. Several studies have already demonstrated the advantages of coupling continuous flow reactors with microwave energy [75,76,77,78].

## 4. Conclusions

A comparison between continuous flow and drop-based microfluidic devices under identical residence times and hydrodynamic and chemical conditions has shown that both approaches exhibit comparable performance in synthesizing AgNPs of similar sizes. This indicates that, under equivalent mixing efficiencies (characterised by consistent Dean numbers), the choice between these microfluidic systems does not significantly impact nanoparticle size.

Despite potential drawbacks such as particle concentration reduction due to deposition or fouling within channels, the continuous flow device is often more practical when evaluating its effectiveness and convenience for further research. This type of device simplifies the process, as nanoparticles do not need to be separated from a separate oil phase. Compared to a drop device, which requires additional steps to construct the PDMS device, operating the entire process is also simplified.

Future research could explore strategies to reduce the deposition of particles within the channels of continuous flow systems, aiming to optimise nanoparticle production efficiency in microfluidic environments. Comparing other synthetic routes in these two types of devices, broadening the size of NPs synthesised, and operating at higher Dean numbers are required to prove whether this similarity in output is of a general nature.

## Figures and Tables

**Table 1:** Size distribution of AgNPs at different flow rates and chemical conditions in the drop reactor.

pH	[TC], mM	Total Flow Rate, μL/min	Average *d* ± *σ*/nm
7	1.91	30	6.86±1.67
7	1.91	15	6.91 ± 1.77
12	1.91	30	3.997 ± 0.73
12	1.91	15	4.51±1.76
7	2.87	30	6.44 ±0.99
7	3.82	30	6.14 ± 1.02

## Data Availability

Data is contained within the article.
